# Solid-State On-Substrate Synthesis of Size-Controlled CuPt@Cu_2_O Core–Shell Nanocubes and Applications for Electrochemical Sensing and Electrocatalytic Methanol Oxidation Reaction

**DOI:** 10.1021/acsami.4c20674

**Published:** 2025-03-13

**Authors:** Louise Colfer, Hazel Neill, Vuslat Juska, Lorraine Nagle, Alan O’Riordan, Nikolay Petkov, Brenda Long, Gillian Collins

**Affiliations:** † School of Chemistry, University College Cork, Cork T12 YN60, Ireland; ‡ AMBER Centre, Environmental Research Institute, University College Cork, Cork, T23 XE10, Ireland; § Tyndall National Institute, University College Cork, Cork T12 R5CP, Ireland; ∥ Centre for Advanced Photonics & Process Analysis, Munster Technological University, Rossa Avenue, Bishopstown, Cork T12 P928, Ireland

**Keywords:** solid-state synthesis, CuPt, alloy nanoparticle, nanocubes, electrochemical sensing, methanol oxidation

## Abstract

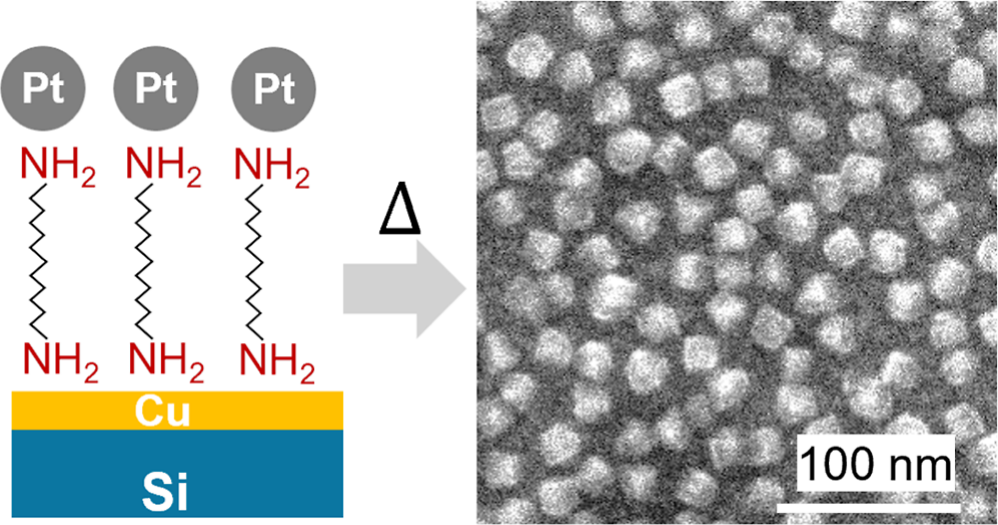

The development of size- and shape-controlled nanomaterials is essential to tailor their properties and performance for wide-ranging applications from catalysis to sensing. Solid-state synthesis of nanostructures is attractive from a sustainability perspective, but they typically lack the desired size and shape control at small-scale dimensions. This work shows that colloidal precursors can be used in a solid-state route to form hybrid core–shell nanostructures with simultaneous size and morphology control. Encapsulation of PtNPs with a well-defined Cu_2_O shell produces CuPt@Cu_2_O core–shell nanocubes grown directly from the underlying substrate. The controlled formation of the nanostructures is facilitated by the diamine passivation layer on the Cu substrate. On-substrate growth of the nanocubes gives ease of postsynthesis processing for them to be used directly in electrochemical applications. We show that the synthesized nanostructured substrates have high sensitivity as an electrocatalyst for glucose sensing. We further demonstrate their potential for direct methanol fuel cells by assessing the methanol oxidation reaction (MOR). The mass activity is determined to be 1.656 A mg_Pt_^–1^ for MOR, and initial studies indicate the substrates show high CO tolerance.

## 1 Introduction

The properties of nanoparticles strongly depend on both size and morphology and so the development of size and shape-controlled core–shell nanostructures are of huge interest due to the enormous potential of these structures toward various applications including catalysis, sensing, energy harvesting, and environmental and biomedical applications.^1−4^ Metal@metal oxide core–shell NPs have attracted immense research interest as the diverse metal-oxide interactions can be used to tune catalytic reactivity, optical and electronic properties, and add stability by suppressing sintering between NPs.^5−8^ To date, the most effective approach for the synthesis of shape-controlled hybrid nanocrystals is through solution-based methods, where it is possible to tailor the exposed facets of crystals, through controlling their nucleation and growth behaviors.^9−12^ The use of seeded growth mechanisms followed by overgrowth of oxide in the presence of a stabilizer has led to the development of complex nanostructures with exceptional simultaneous size and shape control.^12,13^

Colloidal synthesis undoubtedly produces high uniformity both on terms of size and shape, but the resulting nanostructures are often heavily passivated with ligands that are highly challenging to remove, without compromising the original size and shape control and stabilizing ligands are often detrimental to applications due to their passivation of the active surface. Colloidal synthesis routes also utilize excess surfactants and reagents, which need to be removed through postsynthesis solvent washing and purification and often require organic solvents, for solubilizing the stabilizing ligands or as high boiling points solvents. Furthermore, solvents generally contribute the vast majority of the total raw material mass in a synthesis; therefore, the ability to synthesize nanostructures at <50 nm scales under solvent-free conditions is highly attractive from a green and sustainable chemistry perspective.

In contrast, solid-state NP synthesis is attractive as it can provide a simple and scalable preparation method; however, solid-state routes typically have inferior size and morphology control compared to solution-based approaches. In recent years, a variety of solid-state strategies have been developed as comprehensively overviewed by Kumar et al.^14^ Buonsanti and co-workers reported elegant work demonstrating solid-state reactions using colloidal nanocrystal precursors, as an approach to synthesize mixed metal oxides and polyelemental NPs.^15^ Mirkin’s group demonstrated tetrahexahedral particles (∼10 to ∼ 500 nm) with bimetallic compositions, that were synthesized by the solid-state reaction using trace elements antimony, bismuth, lead, or tellurium to stabilize high-index facets.^16^ These findings illustrate the potential of exploring solid-state reactions using nanoparticle precursors.

**Figure 1:**
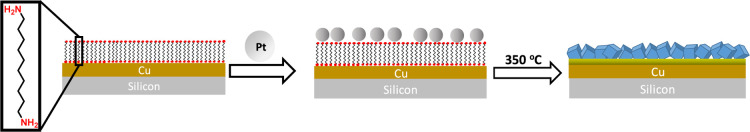
Schematic showing the synthesis steps of the formation of substrate-immobilized PtCu@Cu_2_O core–shell nanocubes.

Here, we report a synthesis method for metal@Cu_2_O core–shell nanocubes using Cu substrates functionalized with a diamine passivation layer to immobilize a high density of PtNPs that can be transformed into CuPt@Cu_2_O nanocubes through annealing under a reducing atmosphere, as illustrated in the schematic in Figure 1. The nanostructured substrate produces well-dispersed nanocubes, which show high uniformity across the substrate and have an edge length of ∼45 nm. The encapsulation of the core with the Cu_2_O shell produces good electrical contact between the nanocubes and underlying substrate. The developed substrate is highly suitable to be used as an electrocatalyst toward glucose detection, and we provide detailed analysis on glucose electro-oxidation via PtCu@Cu_2_O substrate. The electrocatalytic activity of the PtCu@Cu_2_O substrate was also evaluated for the methanol oxidation reaction (MOR). The mass activity for MOR exceeds that reported for commercial Pt catalysts, and the substrates show good tolerance toward catalyst poisoning from CO.

## 2 Results and Discussion

**Figure 2:**
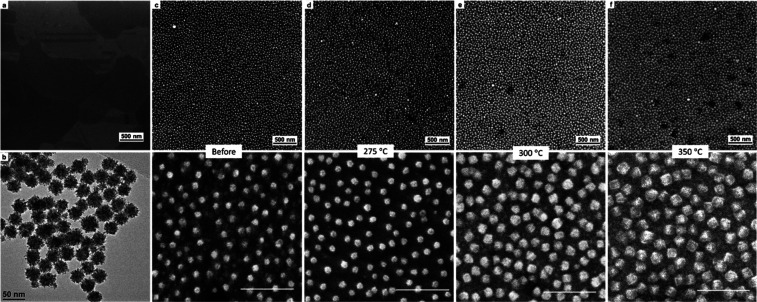
SEM of (a) DAD-functionalized Cu substrates after oxide removal with citric acid. (b) TEM image of citrate-stabilized PtNPs. (c) SEM image of functionalized substrates after deposition of 28 nm PtNPs preanneal and after annealing under H_2_/Ar at a temperature of (d) 275, (e) 300, and (f) 350 °C. Scale bars in figures are 100 nm.

Figure 1 shows the synthesis steps in the formation of the CuPt@Cu_2_O core–shell nanocubes. The first step for the diamine functionalization layer on Cu was removing the native surface oxide without roughening the surface of the Cu substrate. A variety of mineral acids,^17,18^ organic acids,^19,20^ and commercial oxide removal solutions are reported to remove surface oxide but their impact on surface roughness is often not reported. We found that dilute aqueous HCl and NHO_3_ are effective in removing surface oxide but also induce surface roughening. For example, a 1 min 0.37% HCl treatment resulted in an oxide-free surface but also roughening of the Cu surface, as shown by X-ray photoelectron spectroscopy (XPS) and AFM analysis (Supporting Information Figure S1). In contrast to mineral acids, organic acids such as citric acid, acetic acid, and oxalic acid were effective at removing surface oxide as confirmed by XPS, but without etching the Cu surface, as evidenced by AFM, as shown in Figure S2 (see Supporting Information); therefore, citric acid solution was used for removal of the native surface Cu oxide. After oxide removal, the substrates were immersed in a 10 mM methanol solution of diaminodecane (DAD). To prevent reoxidation of the Cu surface, it was important to continually bubble N_2_ through the solution. Heating the DAD solution has been reported by Yamada et al.^21^ to increase the reproducibility and reduce defects in organic monolayers, however heating the solution to 50 °C increased surface roughness of the Cu, therefore the functionalization was carried out at room temperature, and Figure 2a shows an SEM image of the Cu substrate after DAD functionalization.

In this study, we chose to use simple citrate-stabilized PtNPs in water to avoid the use of surfactants or polymers often used in colloidal synthesis. Figure 2b shows a TEM image of the citrate-stabilized PtNPs which consist of a dendritic morphology and have a mean diameter of 28 nm (see Supporting Information Figure S3 for a diameter size distribution histogram). A high density of PtNPs were immobilized without aggregation onto the functionalized surface, as illustrated in the SEM image in Figure 2c. Optimized conditions for this process were found to be 15 min of immersion time in the PtNP solution at room temperature while continually bubbling the solution with N_2_. The Cu surface showed signs of damage with immersion times longer than 15 min, likely due to the slightly acidic citrate NP solution (pH ∼ 6).

Figure 2d–f shows SEM images of the substrates after being annealed under H_2_/Ar at different temperatures. At 275 °C, the PtNP appears relatively unchanged, while increasing the temperature to 300 °C results in the conversion of the immobilized PtNPs into well-defined nanocubes, with a noticeable increase in the size of the particles due to the oxide shell formation. Similarly, nanocube features were formed after annealing at 350 °C, while higher anneal temperatures (>400 °C) resulted in dewetting of the Cu substrate.

The method reported here for the formation of core–shell nanocubes was also applied to smaller diameter PtNPs to assess if this solid-state approach could be used to produce differently sized core–shell nanocubes. TEM images and size distribution analysis of PtNPs with a mean diameter of 13 and 5 nm are shown (see Supporting Information Figure S3). The 5 nm PtNPs could be deposited with high coverage, but some aggregation on the substrate was observed after annealing. The 13 nm PtNPs displayed high coverage on the DAD-functionalized Cu substrate, similar to the 28 nm PtNP (see Supporting Information Figure S4). On annealing the substrate, core–shell nanocubes with uniform dispersion were obtained and are illustrated in the SEM images (Figure S5), demonstrating that this method is applicable using different sizes PtNPs.

SEM analysis of the PtNP-decorated substrates after annealing evidence the encapsulation of the PtNPs in a copper oxide shell to produce well-dispersed nanocubes, which show high uniformity across the substrate and have an edge length of ∼45 nm. The XRD pattern before annealing of the substrate, as shown in Figure S6, shows a strong peak at 43.4° corresponding to the (111) plane of the Cu substrate and a smaller peak at 50.3° corresponding to the (200) plane. After annealing an additional diffraction peak is observed at 36.1°, in good agreement with the (111) planes of Cu_2_O.^22^ There is a small shift in the dominant Cu(111) peak position, which may be due to the influence of Cu_2_O(200) found at 42°. While the XRD analysis suggests the formation of Cu_2_O, a definitive assessment is challenging as the nanocubes are grown on the Cu substrate.

**Figure 3:**
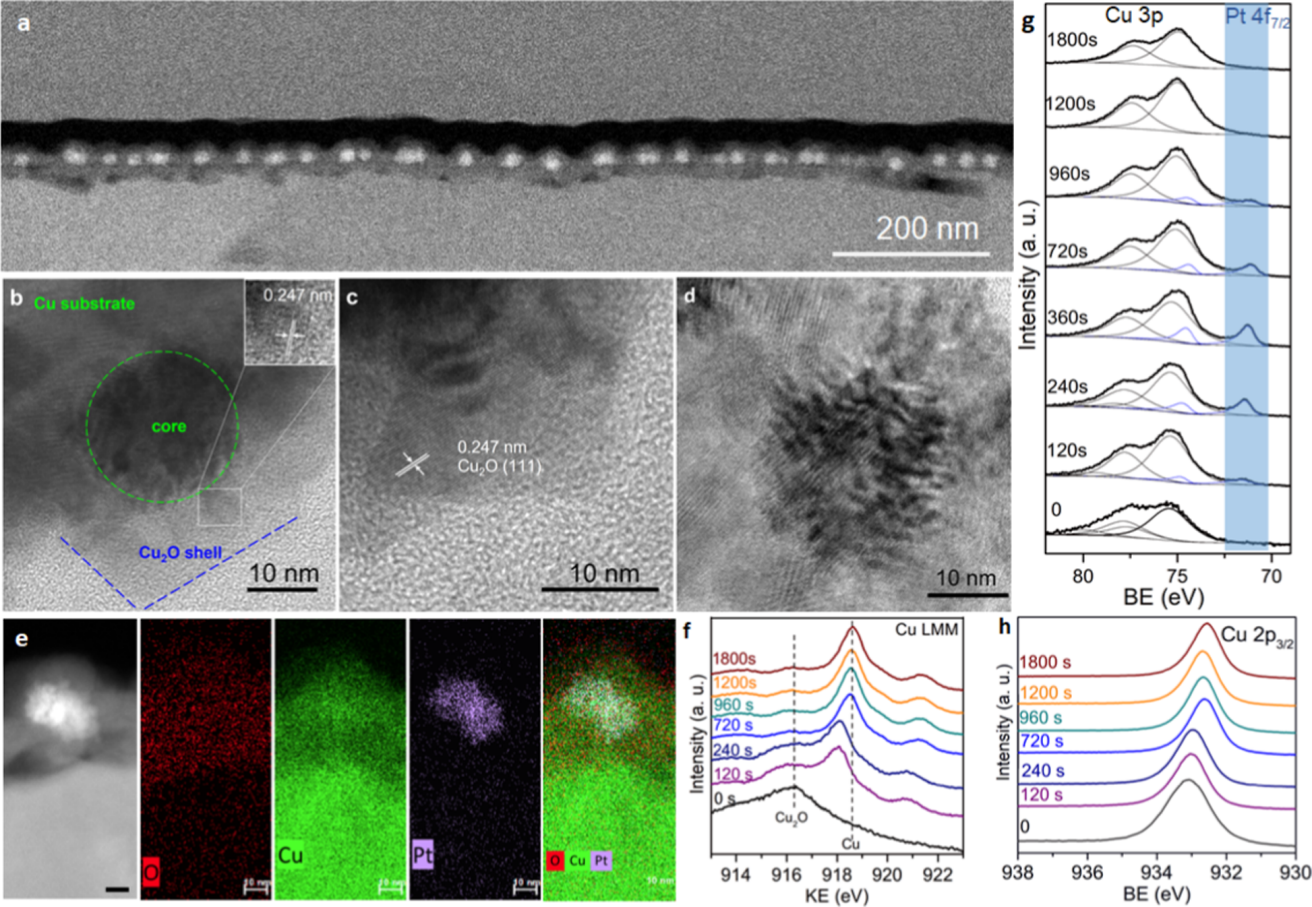
(a) Cross-sectional STEM of the substrates, (b–d) cross-sectional TEM images, and (e) EDX mapping of the core–shell nanocubes. Depth profile XPS analysis of substrates showing the (f) Cu LMM Auger, (g) Cu 3p and Pt 4f, and (h) Cu 2p_3/2_ spectral regions.

XPS analysis and interpretation of Cu–Pt systems are challenging due to the significant overlap with the Pt 4f and Cu 3p region. XPS analysis of the Pt 4f_7/2_ and Cu 2p region of the Pt-decorated substrate is shown in Supporting Information Figure S7. Before annealing, Pt 4f_7/2_ peak can be seen at a BE of 71.3 eV and after annealing the Pt signal is absent, attributed to attenuation of the Pt electrons after encapsulation by the oxide shell due to encapsulation with the Cu_2_O. Due to the small size of the nanostructures and their adherence to the substrate, cross-sectional scanning transmission electron microscopy (STEM) and energy-dispersive X-ray spectrometry (EDX) analysis coupled with depth profile XPS analysis was used to further study the morphology and the composition of the cubic nanostructures formed on the substrates. Figure 3a shows a cross-section STEM image of the nanocubes, which form a well-defined monolayer of NPs across the substrate. High-resolution TEM of the core–shell cross sections, as shown in Figure 3b–d, illustrates the presence of a polycrystalline shell surrounding the metal core. The d spacing of the oxide shell was determined to be 0.247 nm, which is in excellent agreement with the (111) lattice spacing for Cu_2_O.^23^ EDX mapping of the cross section, as shown in Figure 3e, reveals that the Pt is confined to the core and indicates the formation of a CuPt core, although a quantitative assessment on an alloy composition was challenging. XPS depth profile analysis of the Cu LMM, Cu 3p + Pt 4f and Cu 2p_5/2_, and spectral region is shown in Figure 3f–h, respectively. As can be seen in the depth profile of the Cu 3p + Pt 4f region, no Pt is detected due to oxide encapsulation. The Pt 4f_7/2_ peak appears through the profile at a BE of 71.4 eV, consistent with metallic Pt^0^, before disappearing at 1800 s, correlating with the EDX finding that the Pt remains confined to the core. XPS analysis is often used to evaluate the formation of alloy nanoparticles as a core-level shift occurs upon alloying due to the change of electron density in d-band states and thereby give rise to binding energy shifts that can potentially reflect the degree of alloying. Significant shifts are not observed with the Pt 4f_7/2_ because Cu does not affect the electron density in Pt 5d states.^24^ Alloying with Pt has been reported to give rise to red-shifts in the Cu 2p core level.^24,25^ While the Cu 2p_5/2_ depth profile in Figure 3h does show a shift to lower BE, as the spectra are collected in the presence of Ar ion clusters, there is too much uncertainty to correlating BE shifts to electronic properties of the NPs.

The initial Cu LMM Auger peak (Figure 3g) has a clear distinctive peak at a kinetic energy (KE) of 916.3 eV in excellent agreement with Cu_2_O.^26^ The depth profile shows the reduction in the Cu_2_O, and the appearance of a peak at 918.5 eV is in excellent agreement with Cu^0^. Peak shifts can be observed in both the Cu LMM Auger peak and the Cu 2p_3/2_ depth profile but it is challenging to make clear correlations to their origin. BE shifts may be associated with size effects, presence of nonstoichiometric oxides, alloy formation, or artifacts arising from the Ar gas cluster during sample analysis. It has been shown that BE and Auger peak positions for Cu are sensitive to interface effects and particle size effects. Cross-sectional TEM and EDX analyses, coupled with depth profiling XPS analysis, show the presence of the metal core, likely CuPt alloy, and a Cu_2_O shell.

In some regards, the core–shell nanostructures share features similar to the encapsulation of metal nanoparticles on reducible oxide supports under reductive conditions as a result of migration of the surface oxide. These oxide overlayers result from strong metal-supported interactions (SMSI),^27^ and numerous phenomena such as wet-chemistry SMSI^28^ and adsorbate-induced SMSI^29^ have been reported. While our system is distinctly different, in that PtNPs are deposited on metal Cu, the PtNPs are not in direct contact with the substrate due to the diamine organic layer, and the end nanostructure has a geometry that is distinct from the NP and with greater thickness than observed with oxide encapsulation associated with SMSI. However, we rationalized that interfacial effects from the diamine layer may play a role in the formation of the cubic nanostructures and minimizing aggregation of the nanostructures. Amines and other N containing species have been used as surface-directing agents in solution synthesis through preferential adsorption on specific surface facets.^30,31^ Reports on solution synthesis routes to shape-controlled Cu_2_O nanocrystals have shown that altering the concentration of the surfactant can have dramatic impacts on the nanocrystal size and shape.^32,33^

**Figure 4:**
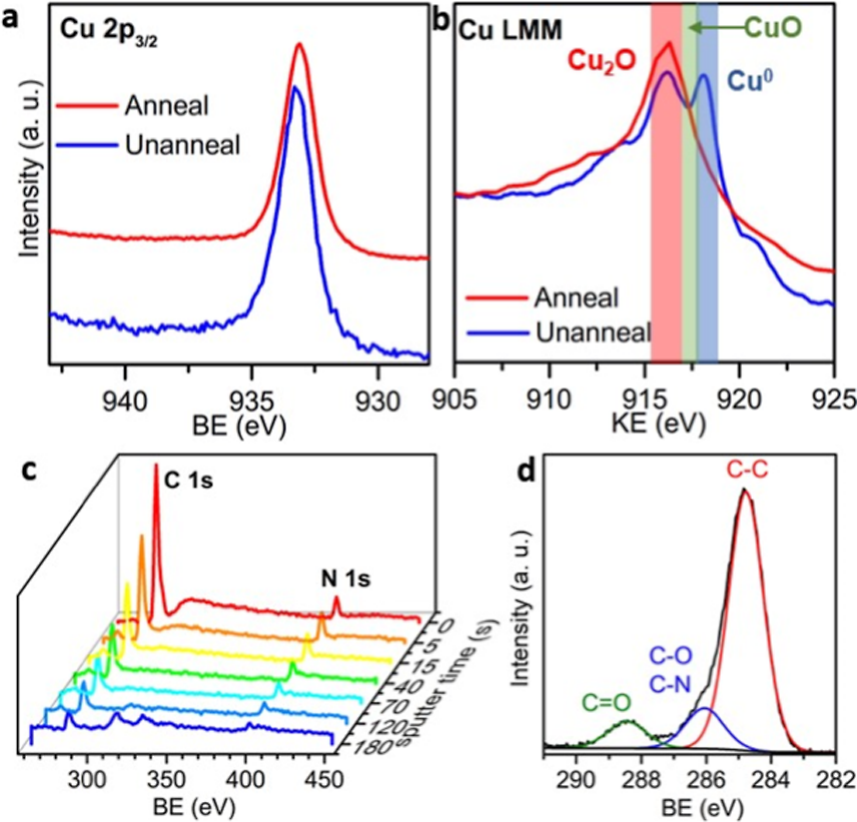
XPS analysis showing (a) Cu 2p3/2 core level, (b) Cu LMM Auger spectra before and after annealing the PtNP immobilized Cu substrates, (c) depth profile XPS of the C 1s and N 1s spectral region, and (d) C 1s core level at grazing angle incidence.

The diamine functionalization layer was used both to passivate the Cu substrate after oxide removal and to anchor the citrate-stabilized PtNPs. Further analysis was carried out to investigate the composition of the interface of the diamine substrates with immobilized PtNPs before and after annealing. XPS survey spectra of Cu substrates at each synthesis step (Supporting Information Figure S8) show the C 1s and N 1s peaks in the diamine-functionalized substrate and the Pt 4f and Pt 3d peaks after PtNP immobilization, as to be expected. Annealing the substrates results in a significant decrease in the intensity of the C 1s signal and the disappearance of the N 1s peak and Pt peaks in the survey. Figure 4a compares the Cu 2p core level of the PtNP-decorated substrates before and after thermal annealing. In the unannealed sample, the dominant Cu 2p_3/2_ peak is located at a BE of 932.9 eV in good agreement with metallic Cu^0^/Cu^1+^ species assigned to Cu_2_O and/or metallic Cu, respectively. There are no significant peaks at 934.9 eV associated with the shakeup satellite attributed to Cu^2+^ species in either sample, indicating CuO is not present, at least to a significant extent.

Metallic Cu cannot be easily distinguished from Cu^1+^ using the 2p core level due to their close BEs, so to discriminate Cu_2_O from Cu, the corresponding Cu LMM Auger peak was evaluated, which is shown in Figure 4b. The broad Cu LMM Auger peak for the unannealed sample exhibits a prominent metallic Cu feature at a KE of 918.1 eV and a peak at 916 eV associated with Cu_2_O.^34,35^ Grazing angle XPS confirms the presence of Cu_2_O in the Cu substrate immobilized with PtNPs. The annealed sample shows a clear decrease in the metallic contribution, which shifts the peak 916.4 eV, again consistent with Cu_2_O oxide shell on the nanocubes. Therefore, the Cu 2p_3/2_ peak at 932.9 eV can be assigned to both metallic Cu from the underlying substrate and surface oxide which is present as Cu_2_O in the PtNP-immobilized substrates prior to annealing. The O 1s core level XPS spectra (Supporting Information Figure S9) of the unannealed substrates, which displays a broad peak (fwhm = 2.4) centered at a BE of 531.3 eV attributed to chemisorbed oxygen species, dissociated oxygen species and water which are typically observed at BE > 531 eV.^36,37^ After annealing, the O 1s peak narrows considerably (fwhm = 1.3) and is downshifted with the peak centered at 530.8 eV, which is in excellent agreement with the formation of Cu_2_O after annealing.^38^

The role of the diamine layer was crucial for the successful formation of the cubic nanostructures. In the absence of a diamine layer, NPs do not uniformly deposit on the Cu substrate when immersed in the PtNP solution. When the PtNPs were spin coated onto the Cu substrate and then annealed, no cubic nanostructures were observed but rather significant surface oxidation occurred in the absence of the diamine passivation layer. When the PtNPs were spin-coated on the Si substrate with a native oxide SiO_2_ overlayer and annealed under H_2_/Ar at 350 °C, to evaluate their stability, no changes to the PtNPs were observed (see Figure S10). The diamine ligands were exchanged for dithiol ligands, and while XPS analysis of the Cu 2p and S 2p confirmed functionalization of the Cu surface, the thiol passivated substrates did not immobilize a high density of PtNPs on the Cu substrates, as shown in the SEM image (Supporting Information Figure S11a–c). Numerous strategies such as altering the solution pH to increase PtNP immobilization were unsuccessful. Studies using AuNPs have shown that citrate species can interfere with adsorption of thiol ligands as hydrogen bonding between citrate species leads to strong adsorption due to the formation of a stabilized network that is challenging to displace.^39^ The ability of the amine group to hydrogen bond with the citrate-stabilized PtNP may play a role in the fast immobilization of the PtNPs (15 min) that was observed on the diamine-functionalized Cu substrates (Figure 4c). XPS depth profile of the N 1s and C 1s core levels reveals residual amines on the surface. Figure 4d shows grazing-angle XPS of the C 1s of the substrates after PtNP deposition, with peaks at 285, 286, and 288.5 eV, associated with C–C, C–O/C–N, and C=O environments. While some C–O and C=O structure will originate from adventitious carbon, the clear peak at 288.5 eV is also associated with residual citrate species, which are known to remain physiosorbed to surfaces when used for oxide removal.^40^ These observations indicate that the citrate species and diamine functionalization layer play a key role in immobilizing a high density of PtNPs and the formation of the cubic nanostructures.

We next explored the potential to extend the solid-state approach to the formation of other metal@Cu_2_O core–shell nanostructured systems. Following the same procedure using the diamine functionalization layer, citrate-stabilized gold (Au) NPs were used instead of the PtNPs. Au@Cu_2_O hybrid core–shell NPs are of interest as they combine the optical signatures of Cu_2_O nanoshells and the plasmonic properties of AuNPs, which can be altered giving plasmonic tunability due to the dielectric properties of the Cu_2_O shells surrounding the Au cores^28^ and gives enhanced photocatalytic efficiency.^41^ Gold@metal oxide core–shell core have been shown to enhance the activity of many 3d transition metal-oxide thin films for the oxygen evolution reaction (OER) in alkaline media.^6^Figure S12 in the Supporting Information shows that the encapsulation of the surface anchored AuNPs to form core–shell structures. The nanostructures clearly increase in size and appear to be faceted, although they do not have a distinct cubic morphology obtained with the PtNPs. While the synthesis parameters of this system were not optimized, and further analysis is required, it nevertheless demonstrates the potential of this solid-state route to be applied to the formation of other metal@Cu_2_O systems.

## 3 Electrochemical Analysis

**Figure 5:**
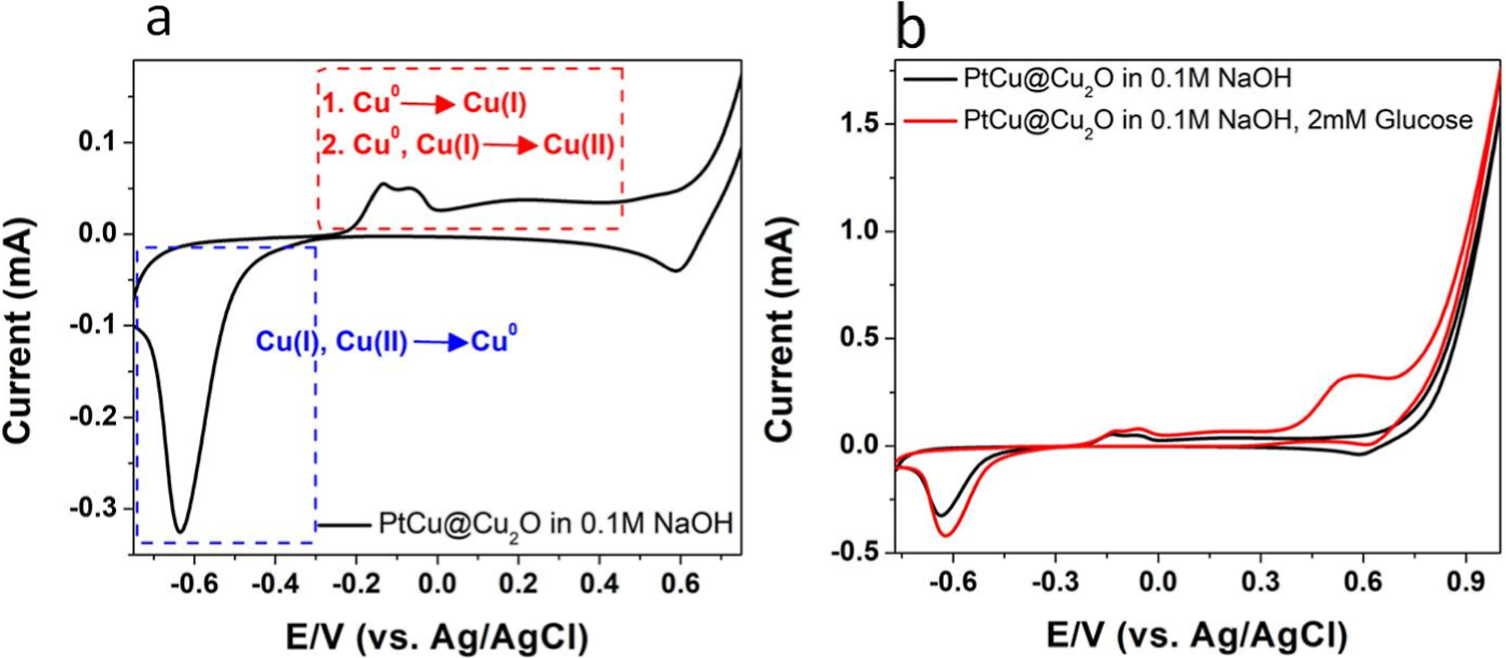
CV of the CuPt@Cu_2_O substrate in 0.1 M NaOH solution; (a) CV of the substrate demonstrating the oxidation of the species and the reduction of the oxidized species; (b) CV of the CuPt@Cu_2_O substrate in the absence and presence of 2 mM glucose.

Typically, electrochemical applications require mixing of NPs with a support material to prepare the working electrode. As the nanocubes are grown from the substrate, they can be used directly without further preparation. In recent years’ noble metal@Cu_2_O NPs, in particular Pt-based NPs have been shown to exhibit excellent electrocatalytic activity toward glucose oxidation, hydrogen peroxide, oxygen reduction, and gas sensing resulting from the electronic and structural effects between core and shell.^42,43^ The as-prepared PtCu@Cu_2_O substrates were used to assess the electrochemical behavior of the surface in an alkaline solution. Figure 5a shows the cyclic voltammogram (CV) of the substrate in a 0.1 M NaOH solution. The multiple peaks starting from −0.2 to +0.4 V are attributed to the oxidation of Cu(0) to Cu(I); then the subsequent formation of Cu_2_O. The following broader peak can be attributed to further oxidation of species of Cu(I) to Cu(II) and Cu(0) to Cu(II). In the reverse scan of the cycle, at around −0.65 V, the reduction peak of the oxidized species can be seen.^44^ After the addition of glucose into the electrochemical cell, a strong oxidation peak is observed at 0.55 V vs Ag/AgCl reference electrode, whereas there was no discernible peak at this potential in the absence of glucose (Figure 5b). The most common hypothesis regarding the glucose electro-oxidation in NaOH via copper oxides involves the proposed formation of Cu(III)-oxides; e.g. CuOOH.^45,46^ While the CV scan was conducted, the outer surface of the substrate undergoes oxidation, resulting in the formation of CuO, which subsequently transitions to Cu^3+^. This Cu^3+^ species is promptly reduced back to Cu(II),^45,47^ and the associated reactions are illustrated in Table S1.^48^

**Figure 6:**
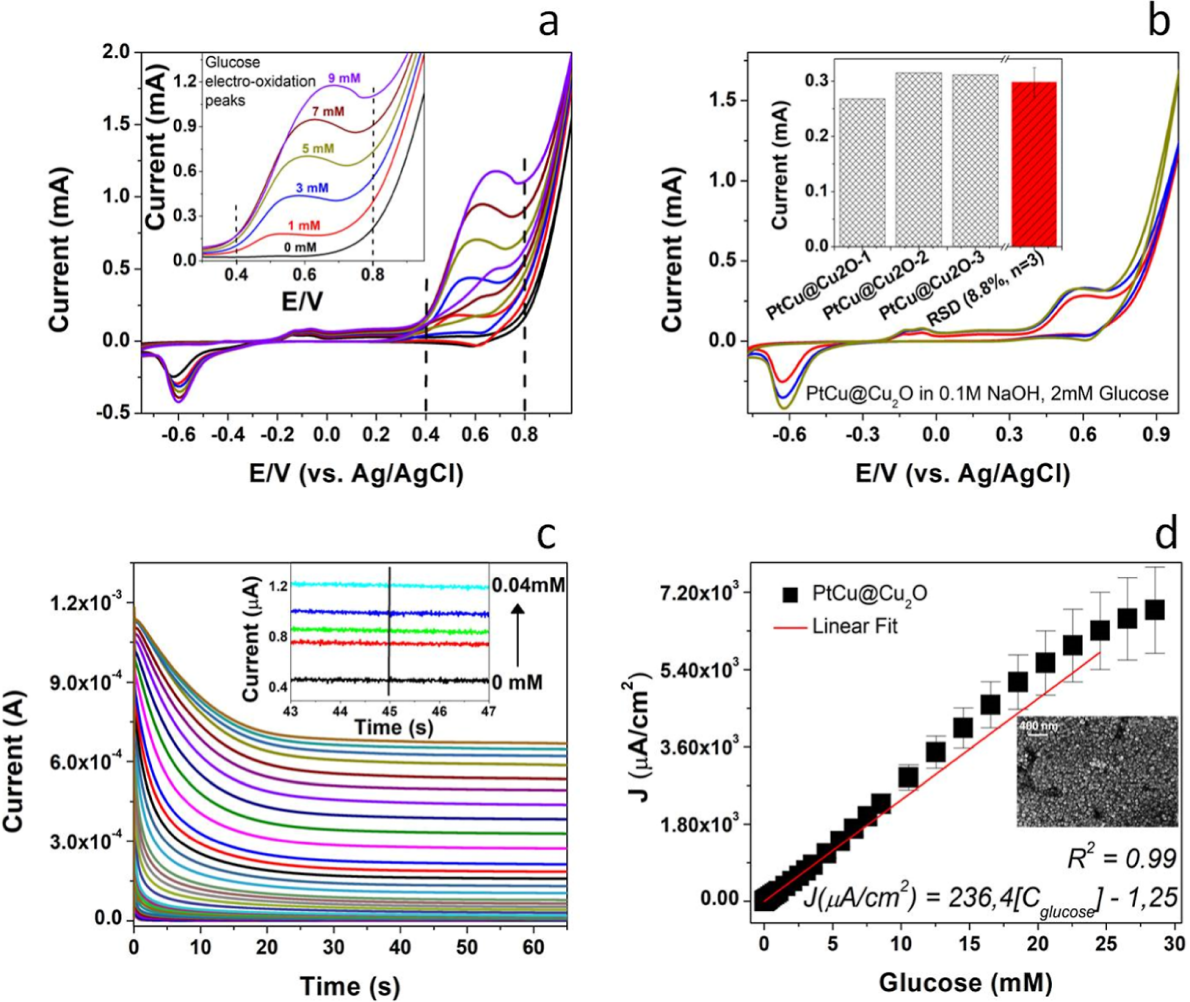
(a) CV showing the effect of increasing glucose concentration. (b) CV assessing the reproducibility of the substrate response. (c) Chronoamperometric responses obtained after each addition of glucose into the electrochemical cell. (d) Assessing the linear concentration range toward glucose.

To analyze the relationship between the increased glucose concentrations and current level, we next tested the substrate with CV (Figure 6a). The anodic peak at +0.55 V increases with the increased concentration of glucose in the electrochemical cell, as shown in the inset graph of Figure 6a. These data indicate that the CuPt@Cu_2_0 is a highly promising candidate as a catalyst toward glucose. The reproducibility of the sensing platform was then investigated, which is a significant parameter for the success of the electroanalysis platform. Figure 6b shows the overlapping voltammograms of three individual substrates measured with 2 mM glucose. The inset graph reflects the oxidation peak currents for each measurement, and the red column represents the calculated average current value (*n* = 3). The obtained relative standard deviation for the reproducibility study is 8.8%. These data clearly demonstrate the success of the direct on-substrate synthesis approach and the high degree of reproducibility of the substrate for electroanalysis.

The oxidation potential determined via cyclic voltammetry, i.e. 0.55 V, was used for the assessment of the electroanalytical performance of the surface for glucose electro-oxidation. Figure 6c shows the raw chronoamperometric responses obtained after each addition of glucose to the electrochemical cell. The inset graph demonstrated the base and the first four additions of glucose with high magnification at 45th second. The current values obtained at 45th second were used to prepare the corresponding calibration curve, as shown in Figure 6d. The PtCu@Cu_2_O substrate exhibited a very wide linear concentration range toward glucose between 0.01 and 24.55 mM glucose. The fitting equation of the PtCu@Cu_2_O substrate was *J* (μA/cm^2^) *=* 236.4 [*C*_glucose_] −1.25 with a correlation coefficient of 0.99. The slope of the calibration range represents the sensitivity of the system, which was calculated to be 236.4 μA mM^–1^ cm^–2^. These results clearly show the advantage of the synthesized substrate in terms of the electro-analytical performance for nonenzymatic glucose sensing. In particular, the wide linear range of the substrate is highly significant since it enables measuring glucose concentrations across a broad spectrum, from low to very high concentration levels, and also minimizes the sample treatment, such as the preparation of the dilutions. For example, Siampour et al.^49^ reported the binder-free Au@Cu core–shell nanostructures for nonenzymatic glucose detection, which demonstrated two linear ranges, i.e., 5 μM–2.1 mM and 2.1–7 mM. Although the first range demonstrated a high sensitivity, it has a very narrow linear range. They documented a sensitivity of 327 μA mM^–1^ cm^–2^ for the second concentration range up to 7 mM, slightly surpassing the sensitivity of our substrate based on PtCu@Cu_2_O which is capable of detecting glucose up to 24.55 mM. We foresee that such substrates can be an excellent candidate for other biosensing platforms such as chemical oxygen demand for environmental analysis^50^ and immobilization matrix for biomolecules for affinity-based sensors^51,52^ or enzymatic sensors.^53^ Furthermore, the PtCu@Cu_2_O substrate can be miniaturized for integration with other components such as microfluidics, which may further enhance its electroanalytical performance.^54^

The unique aspect of the synthesis method demonstrated in this work is the formation of size-controlled nanostructures grown directly from the substrate. In this context, developing the Pt-based nanoalloys has attracted much interest as a route to raising the efficiency of Pt atoms and decreasing catalyst price for a range of catalyst applications.^55−57^ Among the Pt-M alloys, PtCu is emerging as a robust electrocatalyst for fuel-cell technologies owing to the inexpensive cost and electrocatalytic activity of copper. The PtCu@Cu_2_O substrate was evaluated for methanol oxidation reaction (MOR) under alkaline conditions to establish if an enhancement in catalytic activity with a substantial reduction in materials cost is feasible while providing a well-defined model system for a future mechanistic study. MOR on Pt follows a parallel pathway mechanism forming several reaction intermediates and products (CO_2_, HCOOH, and HCHO) as well as the insidious active site-blocking CO, which are illustrated in Supporting Information Table S1.^58^ The active site may constitute low coordination number atoms present at steps, grain boundary, and defect sites.^59^

The combination of Pt with other metals can overcome the stability issues. For example, improving CO tolerance in MOR at low potentials is challenging as its oxidation to CO_2_ requires oxygen atoms, which is typically facilitated via the dissociation of water, yet Pt only chemisorbs OH above 0.7 V. Alloying Pt with other oxophilic metals which adsorb OH at lower potentials, such as Ru, Ir, Rh, Os, Cu, and Sn, can reduce CO poisoning and increase catalytic activity in a bifunctional mechanism.^57,60^ This enhanced activity of Pt alloys can also relate to a modification of the Pt electronic structure with a weakening of the Pt–CO binding strength. Maturost et al.^61^ reported a nanocomposite catalyst consisting of multiwall carbon nanotubes, CuO, and PtNPs for MOR and found that CuO reduces CO adsorption on the catalyst surface through a significant adsorption of hydroxyl species that can exclusively transform CO to CO_2_ to leave behind accessible, active Pt catalyst surfaces.

**Figure 7:**
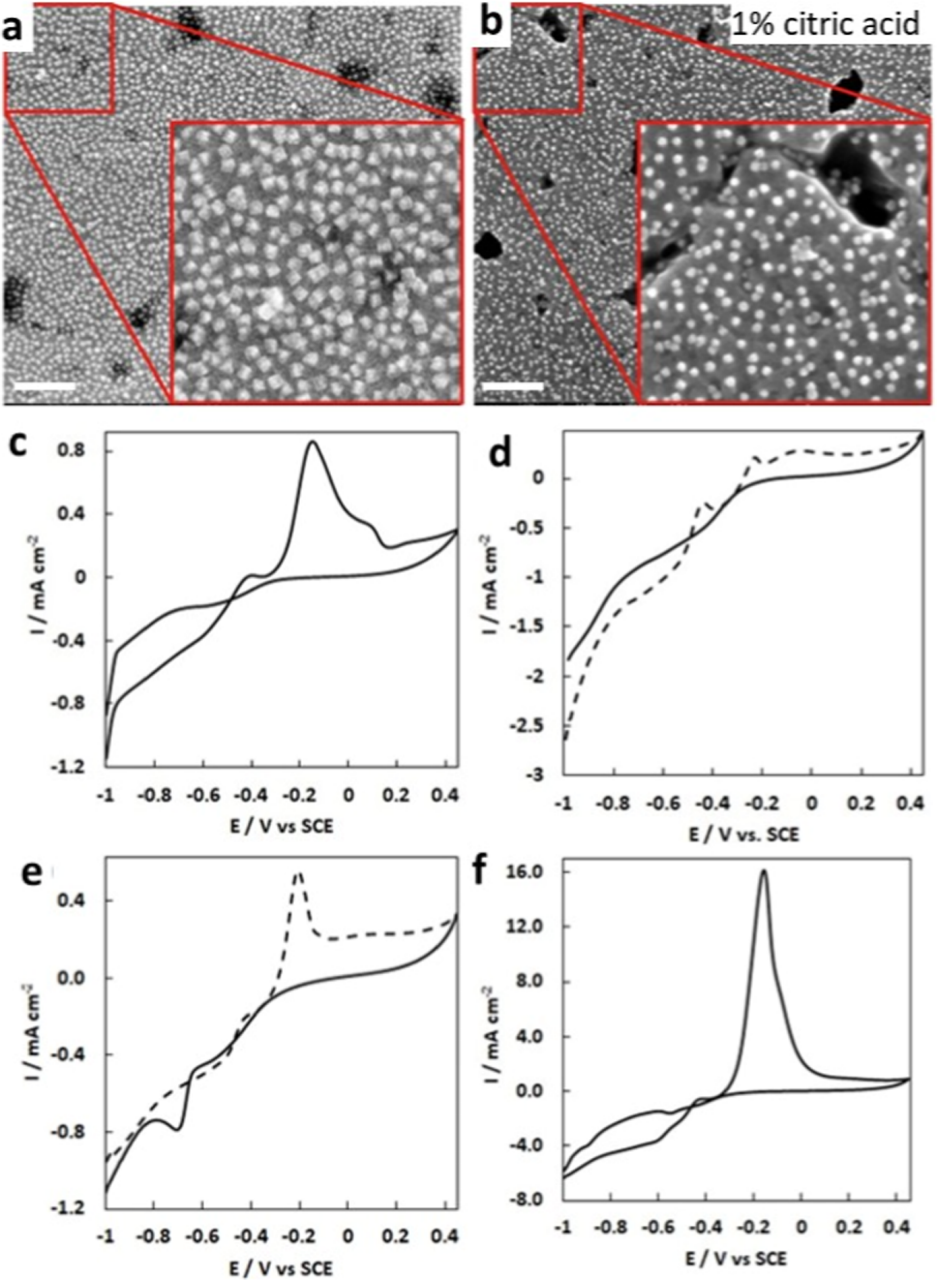
SEM image of (a) as-prepared PtCu@Cu_2_O nanocube substrates and (b) after etching with 1% citric acid (scale bars are 100 nm). (c) CV for PtCu@Cu_2_O nanocube array in 1 M KOH, (d) CV for PtCu@Cu_2_O nanocube array in 1 M KOH after citric acid etch, (e) CV for PtCu@Cu_2_O nanocube array in 1 M MeOH in 1 M KOH, and (f) CV for PtCu@Cu_2_O nanocube array in 1 M MeOH in 1 M KOH after citric acid etch.

As the cross-sectional TEM analysis shown in Figure 3, revealed adhesion of the NPs to the underlining substrate, we investigated the removal of the Cu_2_O shell to expose the PtCuNPs embedded in the Cu substrate. The sample was etched in 1% citric acid for 90s to remove the Cu_2_O shell, as shown in Figure 7a,b. The CVs of the substrates were recorded in Ar-degassed 1 M KOH in the absence and presence of methanol and are shown in Figure 7c,d, respectively. The as-prepared substrate was subjected to three CV scans in 1 M KOH prior to recording its response for MeOH. In Figure 7c, the oxide peaks at −0.40, −0.12, and −0.10 V may be assigned to the oxidation of Cu(0) to Cu(I), Cu(I) to Cu(II), and Cu(0) to Cu(II), respectively. In Figure 7d, the Cu(0) to Cu(II) oxidation peak is absent, and the former two are hugely diminished. It is possible that their presence, albeit greatly reduced, represents the oxidation of the underlying copper support to Cu_2_O and CuO. The oxidation current in the region −0.2 to 0.4 V is indicative of platinum oxide formation; however, the expected reduction peak at ca. −0.30 V may be masked by the reduction current associated with the copper and/or hydrogen evolution. Figure 7e shows the CVs in Ar-degassed 1 M KOH in the absence of MeOH, and Figure 7f shows the CVs in 1 M KOH and 1 M methanol, both at a scan rate of 20 mV/s. Significant activity for MOR is observed after removal of the Cu_2_O shell, and a large oxidation peak for MeOH is seen in the forward anodic scan at −0.185 V. In the reverse scan, the complete absence of an oxidation peak is noted. This demonstrates that MeOH was effectively oxidized without the generation of the poisoning intermediates. It is plausible that the copper support could act as a co-catalyst as its oxidation generates CuO and/or Cu_2_O (at potentials below the onset for MeOH oxidation) which can assist in the oxidative removal of CO_ads_ given their oxophilic nature such that MeOH is oxidized to CO_2_. Halim et al.^62^ reported that Cu_2_O–Cu(OH)_2_ nanodendrites on carbon nanofibers/poly(paraphenylenediamine) nanocomposite displayed high catalytic activity and stability for MOR under alkaline conditions with the absence of an oxidation peak in the reverse CV, indicating good tolerance of Cu_2_O and/or Cu(OH)_2_ toward catalyst poisoning from CO. Rod-shaped Cu_2_O NPs were reported to oxidize MeOH completely to CO_2_ and H_2_O, displaying no oxidation peak in the reverse scan, again highlighting the potential of Cu_2_O as a MOR electrocatalyst.^63^

Based on the Pt mass loading of 9.6 μg/cm^2^ used herein, the mass activity for MOR is 1.656 A mg_Pt_^–1^. This exceeds that reported for commercial Pt catalysts composed of 20 wt % Pt on Vulcan carbon XC-72 (0.300 A mg_Pt_^–1^).^64^ A comparison of the MOR performance of our PtCu nanocube array with Pt-based electrocatalysts recently reported in the literature is shown in Table S2 and our catalyst outperforms several of those listed. The mass activity exceeds that for PtCu mesoporous nanowire catalysts (0.741 A mg_Pt_^–1^)^64^ and Pt_3_Cu nanodendrites on TiO_2_ (0.437 A mg_Pt_^–1^),^65^ which were evaluated in 1 M MeOH, 1 M KOH, and 0.5 M MeOH in 0.5 M H_2_SO_4_, respectively.

Direct comparison of our catalyst’s performance for MOR with that of other PtCu nanocube catalysts reported in the literature was not possible as their mass activities were not stated, just current densities, yet our findings concur with those reported for this particular morphology of PtCu nanoparticles.^66,67^ Xu et al.^66^ showed that solution-synthesized PtCu nanocubes deposited on the glassy carbon electrode demonstrated superior electrocatalytic activity to PtCu nanospheres and Pt nanospheres which they attributed to the electronic structure modification of Pt in the presence of Cu and to (100)-terminated PtCu nanocube morphology possessing a higher MOR activity than others with mixed crystallographic facets. Wang et al.^67^ reported that PtCu nanocubes immobilized on graphene exhibited better MOR activity and stability than PtCu hexagon nanosheet, PtCu nanoellipsoid, and commercial Pt/C in alkaline medium.

While the MOR study presented is preliminary, it demonstrates the potential for this substrate synthesis method for use in such applications. Undoubtedly, a more detailed and comprehensive study of MOR at these substrates is warranted in future work, particularly an evaluation of its resistance to CO poisoning. The inclusion of Cu atoms with a smaller lattice constant into Pt (lattice constant for Pt and Cu are 3.92 and 3.62 Å, respectively) leads to a broadening of the Pt d-band and downshift of its band center which weakens the CO adsorption strength. In addition, Cu can absorb oxygenated species for CO oxidation at lower potentials than Pt, thus facilitating its removal.^68^ Future CO stripping experiments will reveal the onset and peak potentials for oxidation of a preformed CO adlayer on the substrate, which can provide an insight into the ease of its oxidation/removal. If a negative shift in the aforementioned potentials for CO is observed at the substrate relative to commercial platinum catalysts, this would indicate a heightened CO tolerance due to a weaker CO binding energy. The substrate’s poison tolerance can be further assessed in chronopotentiometric analysis at a fixed current density. Determination of the electrochemically active surface area (ECSA) for the substrate as well as Tafel slope values obtained from linear sweep voltammetry, chronoamperometry, and EIS will be carried out. An investigation into which of the parallel pathways leads to MOR would also be an insightful follow-on study. The indirect (CO) pathway proceeds via serial dehydrogenation steps to form CO, which can subsequently be oxidized to form CO_2_, while the direct (non-CO) one involves CHO hydroxylation producing HCOOH species, which can form CO_2_ via serial dehydrogenation steps. The substrate may provide an abundance of active sites favoring the non-CO pathway. The substrate’s durability is also important for applications and will be examined in future work using chronoamperometry at a fixed voltage and morphology retention which may influence the alloys catalyst’s stability.^69^ In summary, albeit preliminary in nature, the initial exploratory electrocatalytic studies nonetheless illustrate the potential of these substrates for MOR.

## 4 Conclusions

This work demonstrates the capability of using colloidal precursors in a solid-state route to form hybrid core–shell nanostructures with simultaneous size and morphology control. The presence of a diamine passivation layer enables a high density of PtNP to be immobilized on the Cu surface which on annealing is converted to a CuPt@Cu_2_O nanocube. This synthesis method poses numerous benefits over traditional colloidal routes such as being solvent free. Cross-sectional TEM analysis showed the nanostructures are in good electrical contact with the underlying substrate, resulting in nanostructured arrays that can be used directly in electrochemical analysis applications. We foresee that such an interface is a promising candidate to explore the electrochemical sensing platforms not only as an electro-catalyst but also as an immobilization matrix for biomolecules attachment. Furthermore, assessment in MOR reveals good catalytic activity and indicates potential synergistic benefits through the oxophilic nature of the underlying substrate which can promote the oxidative removal of CO_ads_, thus minimizing the poisoning effect of active sites on Pt. Indeed, the MOR may also have proceeded through the formation of HCOO^–^ as an intermediate product, resulting in CO_2._ While a comprehensive study of the electrocatalytic performance and mechanistic study is required, we believe this work will encourage further investigation into exploring solid-state reactions using NPs as precursors for the formation of hybrid core–shell nanostructures.

## 5 Experimental Section

### 5.1 Chemicals

Potassium tetrachloroplatinate (K_2_PtCl_4_), sodium citrate, citric acid, l-ascorbic acid, sodium borohydride NaBH_4_, 1,10-diaminodecane, sodium hydroxide (NaOH), glucose, methanol, isopropyl alcohol (IPA), and acetone were purchased from Sigma-Aldrich. The Cu substrates consisted of a Cu layer prepared by chemical mechanical polishing with a thickness of ∼ 100 nm deposited on Si substrates with a TaN adhesion layer.

### 5.2 Synthesis of Pt Nanoparticles

Synthesis of PtNP seeds: the Pt nanoparticles were grown via an aqueous phase seed-mediated method using a modified literature procedure.^70^ Briefly, 46.4 mL of deionized (DI) water and a stir bar were added to a two-neck 100 mL round-bottom flask, equipped with a reflux condenser, and heated to reflux. To this was added 3.6 mL of a solution containing 0.2% (w/v) K_2_PtCl_4_ solution. After 1 min, 1.1 mL of a solution containing 1% (w/v) sodium citrate and 0.05% (w/v) citric acid solution was added to the round-bottom flask. This was followed 30s later by an addition of 0.55 mL of freshly prepared NaBH_4_ (0.08%) solution, containing 1% sodium citrate and 0.05% citric acid. The round-bottomed flask was then stoppered, and the solution was allowed to reflux for 10 min and then left to cool to room temperature.

Synthesis of 28 nm Pt NPs: for the seeded synthesis, 29 mL of DI water and 1 mL of the PtNP seed solution were added to a two-neck round-bottom flask equipped with a condenser and magnetic stir bar. Depending on the desired nanoparticle size, a different quantity of the 0.4 M Pt precursor solution (K_2_PtCl_4_) was added. To synthesize ∼10 nm NPs, 10 μL of the Pt precursor solution was added, and in the case of ∼ 30 nm NPs that value was 45 μL. After addition of the Pt precursor, 0.5 mL of a solution containing 1% (w/v) sodium citrate and 1.25% (w/v) l-ascorbic acid was added to the flask. The solution was heated to reflux, aged for 20 min, and then left to cool to room temperature. The nanoparticles were collected by a centrifuge and washed three times with water.

### 5.3 Pt NPs Deposition on Surface Functionalization of Cu Substrates

The substrates were cut to the desired size of approximately 1 × 1 cm. The substrates were degreased by sonicating in acetone for 3 min. The substrates were removed from the acetone and washed with IPA and dried with N_2_. The native surface oxide was removed by immersing the substrate into a 1% (w/v) % citric acid solution (degassed with N_2_), for 10 min while continually under a flow of N_2_, via a syringe. The substrates were removed and placed into a vial containing a 10 mM 1,10-diaminodecane solution in methanol for 24 h. N_2_ gas was continually bubbled through the diamine solution. After 24 h, the substrates were removed and placed in N_2_ degassed methanol for 10 min. They were then removed, washed with IPA, dried with N_2_, and immersed in the PtNP solution, which was continually bubbled with N_2_. After 15 min, the substrates were removed, dried with a N_2_ gun, and transferred into a reaction cell for annealing. In a typical annealing experiment, the reaction cell was placed in the tube furnace and annealed under 5% H_2_/Ar at temperatures ranging from 250 to 500 °C for a period of 30 min. After which time, the tube furnace was turned off and the reaction cell was allowed to cool to room temperature before disassembling and removing the substrates.

### 5.4 Materials Characterization

Scanning electron microscopy (SEM) was carried out using a Zeiss Supra 40 instrument. Transmission electron microscopy (TEM) of the Pt NPs was performed on a JEOL 2100 LaB6 instrument at an operating voltage of 200 kV. Cross-sectional STEM and EDX were performed on a FEI Titan with an operating voltage of 300 kW. Depth profile XPS was collected and acquired using a KRATOS AXIS 165 monochromatized X-ray photoelectron spectrometer equipped with an Al Kα (*h*v = 1486.6 eV) X-ray source. Spectra were collected at a takeoff angle of 90, and all spectra were referenced to the C 1s peak at 284.8 eV. TEM and STEM analyses were performed using a FEI Titan TEM, at an operating voltage of 300 kV.

### 5.5 Electrochemical Analysis

#### 5.5.1 Electrochemical Detection of Glucose

All electrochemical studies were performed at room temperature with an Autolab potentiostat (PGSTAT-302N, Metrohm-UK). Electrochemistry of the materials was evaluated in a three electrode system which consisted of a working electrode, a Ag/AgCl (1 M KCl) reference electrode, and a spiral-Pt counter electrode in a 10 mL solution volume. As the working electrode, the synthesized substrate was used. The cyclic voltammetry was studied to characterize the material in 0.1 M NaOH solution with an applied scan rate of 0.1 V between a voltage range from −0.8 V to +1.0 V. The chronoamperometry was applied to investigate the electro-analytical performance of the substrate. For this purpose, we applied a voltage of 0.55 V (vs Ag/AgCl) for 65 s. The calibration study was obtained with the chronoamperometric measurements of subsequent additions of 10 μL glucose solutions into the electrochemical cell. Relative standard deviations (RSDs) were calculated as follows: RSD %= (σ/μ)x100, (*n* ≥ 3), where σ is the standard deviation and μ is the mean of the limiting currents. The equations for the linear range and corresponding slope values were calculated by using Origin2020 software.

### 5.6 Methanol Oxidation Reaction Measurements

Electrochemical analysis was performed using a potentiostat (MAC80150 Autolab, Utrecht, The Netherlands) and a Faraday cage connected to a PC. A three-electrode electrochemical cell was implemented; using PtCu@Cu2O core–shell nanocube substrate as working electrodes, platinized-titanium mesh as a counter electrode, and a double junction Red Rod REF201 standard calomel electrode supplied by Radiometer as a reference electrode. Prior to all electrochemical measurements, electrodes were cleaned in acetone, IPA, and DI water and allowed to dry in air.

## Supporting Information Available

XPS and AFM analyses of before and after surface oxide removal using citric acid and HCl; TEM and size distributions of PtNPs; SEM images of DAD-functionalized Cu substrates after immobilization of PtNPs; SEM images of nm PtNPs deposited on substrates annealed at different temperatures; XRD of substrates before and after annealing; Pt 4f and Cu 3p spectral region of substrates before and after annealing; XPS survey and O 1s scans; PtNPs spin-coated onto the SiO_2_ substrate; XPS and SEM analyses of substrates using dithiol in place of diamine; SEM of AuNP on Cu substrates; proposed reactions in Pt-catalyzed glucose electro-oxidation in NaOH and MOR under alkaline conditions; and mass activity of nanocatalysts reports from literature (PDF)
