# Supplementary material for: Solid-State On-Substrate Synthesis of Size-Controlled CuPt@Cu2O Core–Shell Nanocubes and Applications for Electrochemical Sensing and Electrocatalytic Methanol Oxidation Reaction
Source: ACS Appl Mater Interfaces. 2025 Mar 13;17(12):18243–54. doi: 10.1021/acsami.4c20674 (PMC11956002; doi:10.1021/acsami.4c20674)
Supplement: Supplementary file 1 — am4c20674_si_001.pdf [file am4c20674_si_001.pdf]

Supporting Information

Solid-state on-substrate synthesis of size-controlled CuPt@Cu_2O core-shell nanocubes and applications for electrochemical sensing and electrocatalytic methanol oxidation reaction

Louise Colfer[1, 3], Hazel Neill[1, 3], Vuslat Juska[3], Lorraine Nagle[3], Alan O'Riordan[3], Nikolay Petkov[3,4], Brenda Long[1,2*] and Gillian Collins[1,2*]

[1] School of Chemistry, University College Cork, Cork, T12 YN60, Ireland.

[2] AMBER Centre, Environmental Research Institute, University College Cork, Cork, T23 XE10, Ireland.

[3] Tyndall National Institute, University College Cork, T12 R5CP, Cork, Ireland.

[4]Centre for Advanced Photonics & Process Analysis, Munster Technological University, Rossa Avenue, Bishopstown, Cork, T12 P928, Ireland.

To whom correspondence must be addressed: email: g.collins@ucc.ie, brenda.long@ucc.ie

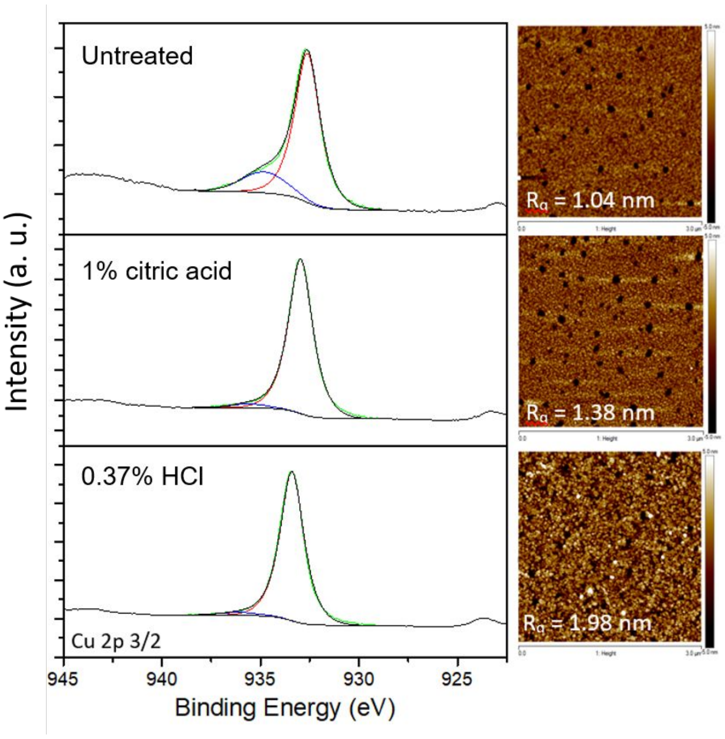

Figure S1: Cu2p$_{3/2}$ od untreated substrate sand after treatment with 1% citric acid for 10 min and 0.37 % HCl for 1 min.

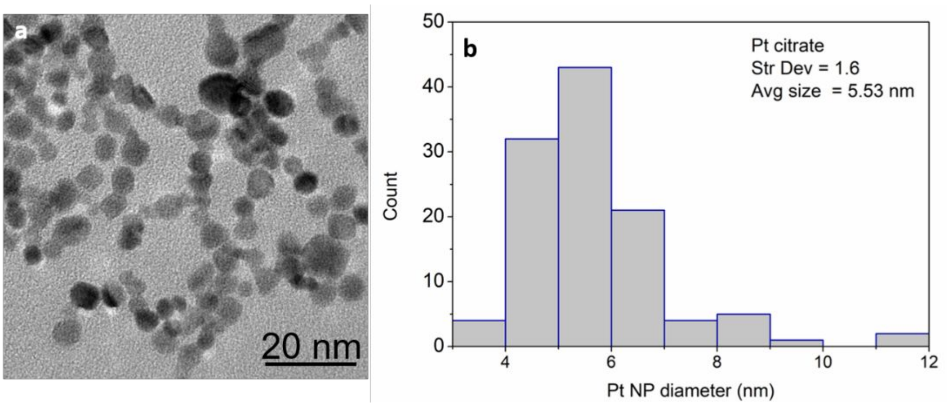

Figure S2: (a) TEM image of citrate stabilised Pt seed NPs and (b) associated size distribution histogram.

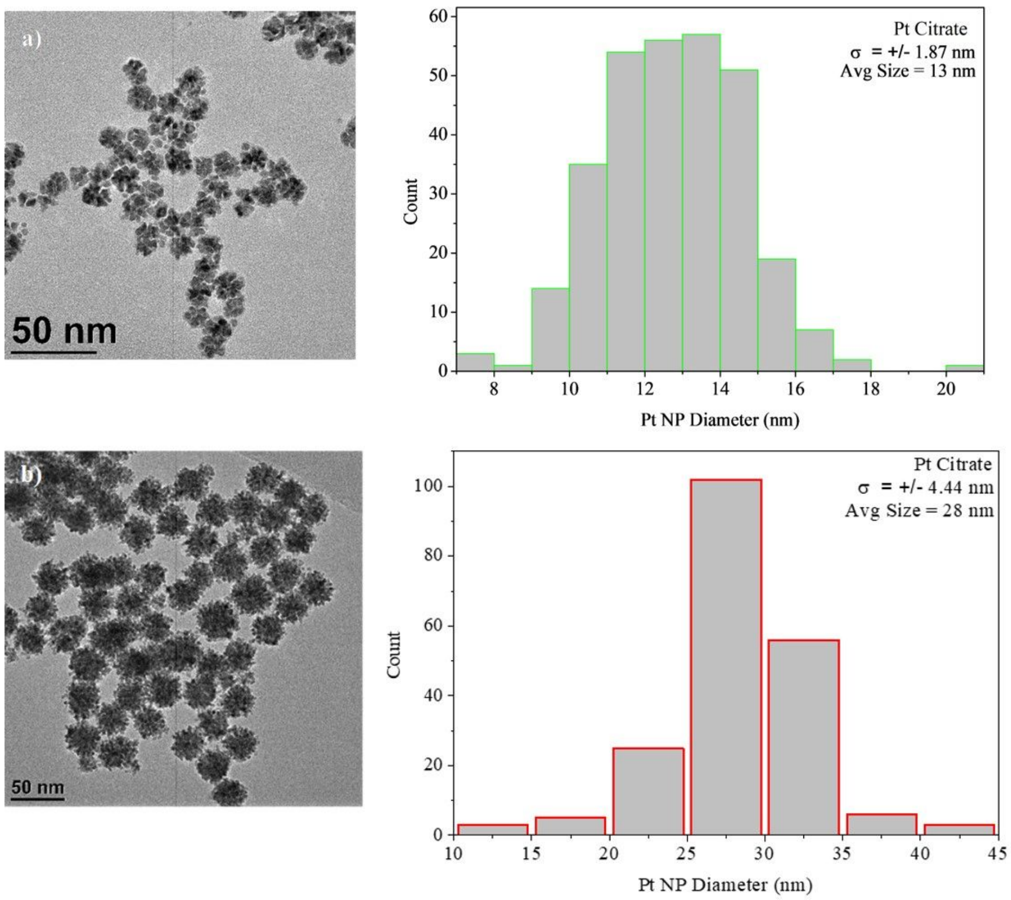

Figure S3: TEM images and associated size distribution histogram of citrate stabilized PtNPs with a mean diameter of (a) 13 nm and (b) 28 nm.

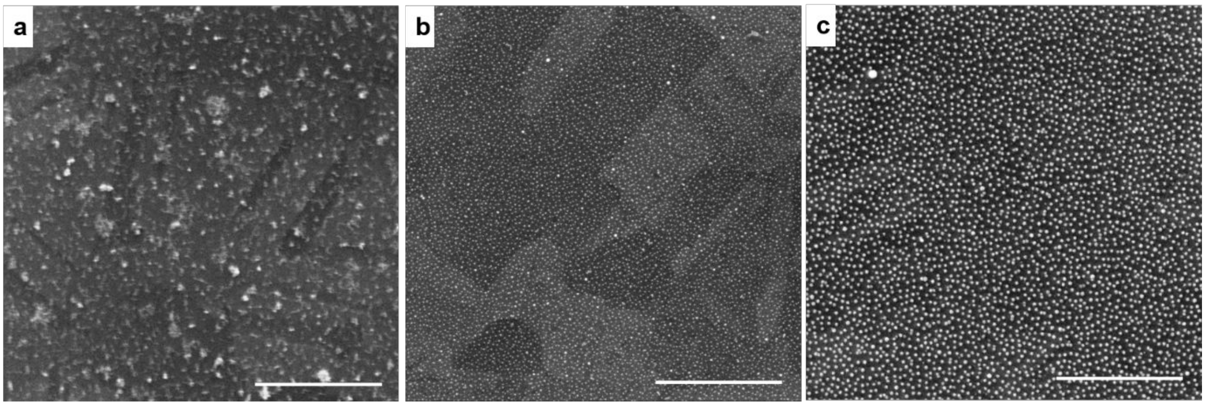

Figure S4: SEM images of DAD-functionalized Cu substrates after immobilization of citrate stabilised PtNPs with mean diameter of (a) 5.5 nm (b) 13 nm and (c) 28 nm.

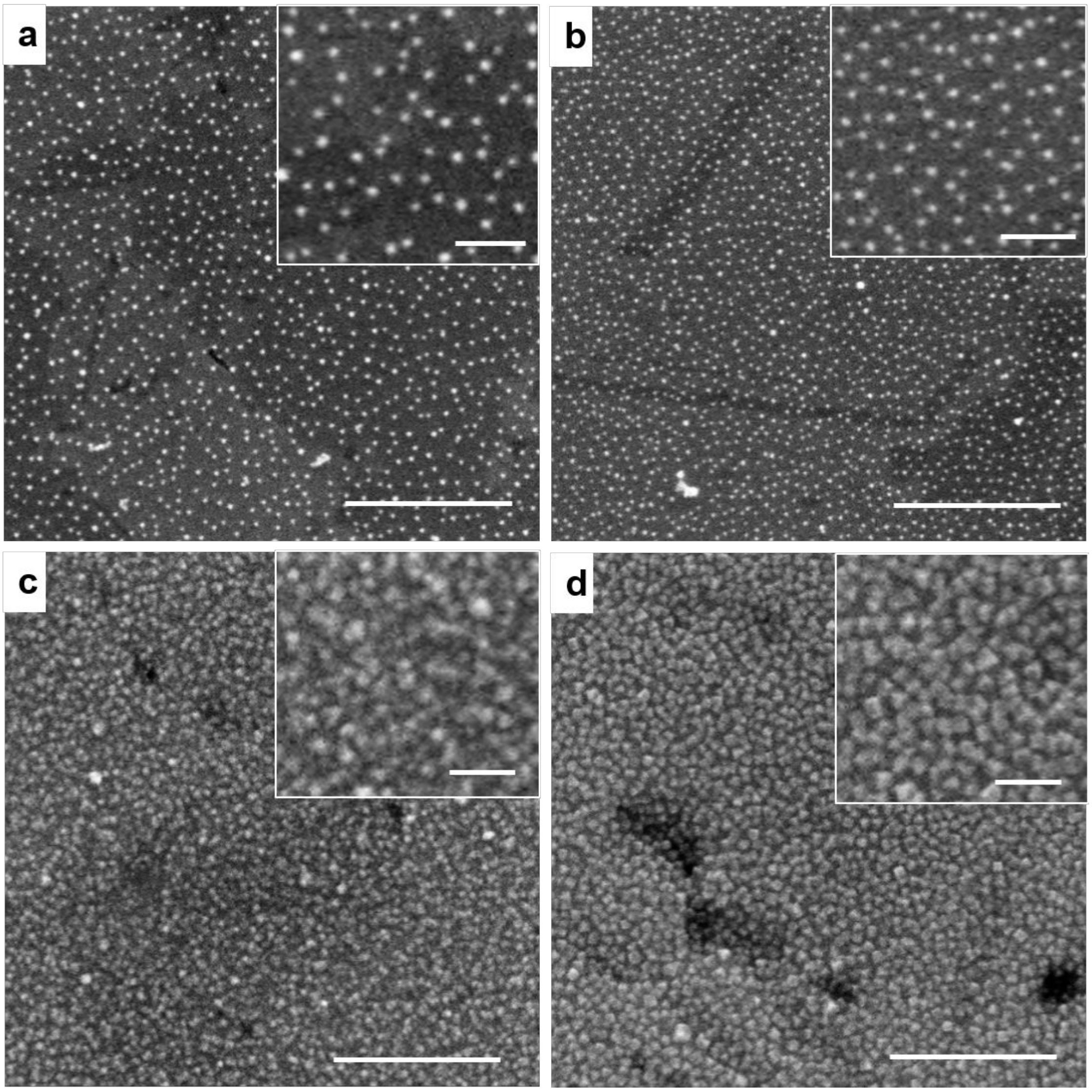

Figure S5: SEM images of 13 nm PtNPs deposited on DAD functionalized Cu substrates (a) pre-anneal and after annealed under H_2/Ar at a temperature of (b) 275°C (c) 300 ^{o}C and d) 350 ^{o}C. Scale bar in main image is 500 nm. Scale bar in figures inset are 100 nm.

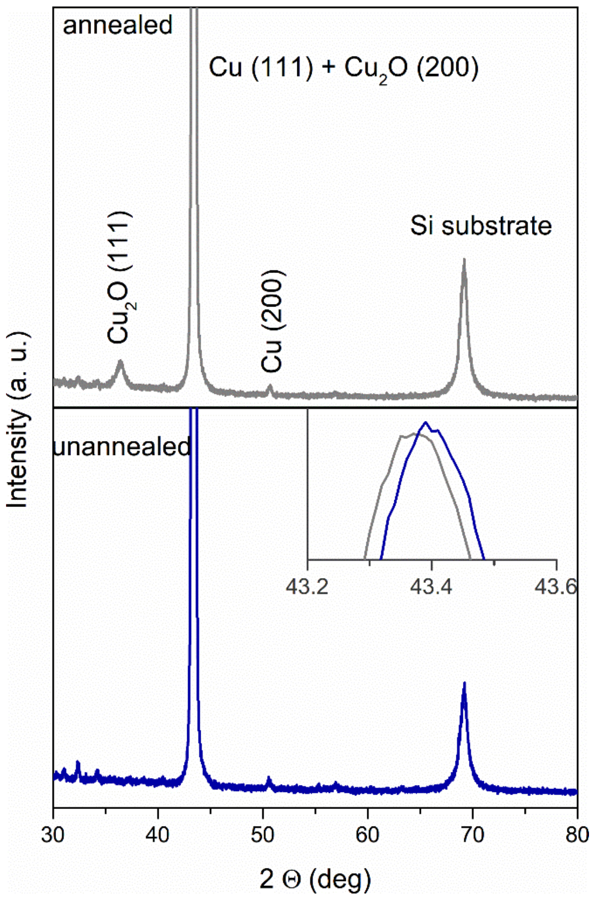

Figure S6: XRD of substrates before and after annealing.

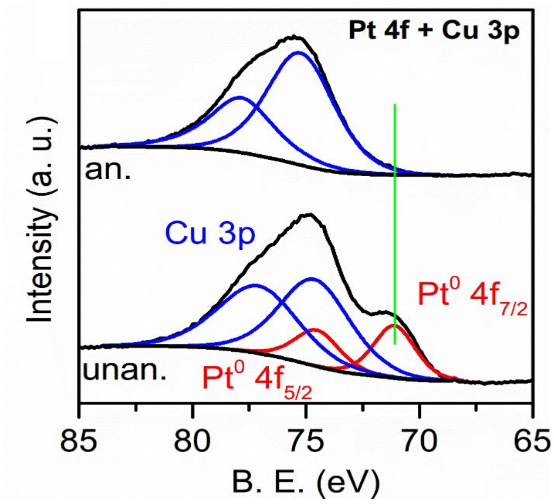

Figure S7: Pt 4f and Cu 3p spectral region of the PtNPs deposited on the functionalized Cu substrates before and after annealing at 350 °C under H_2/Ar.

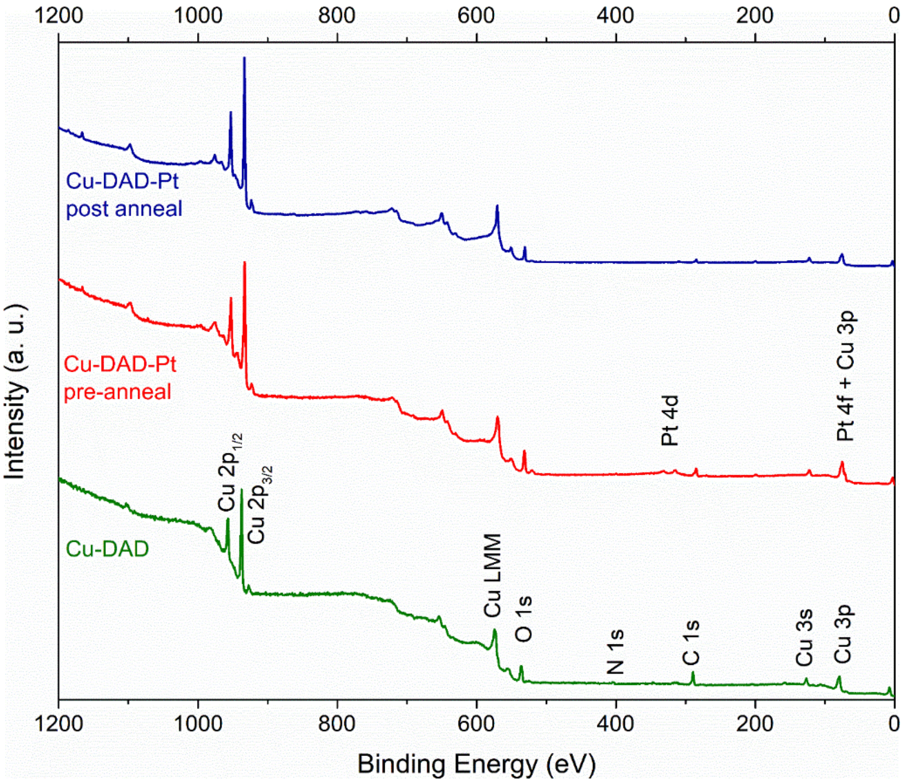

Figure S8: XPS survey scans of Cu substrate functionalized with diamine (DAD), after Pt NP deposition and after annealing at 350 °C under H_2/Ar.

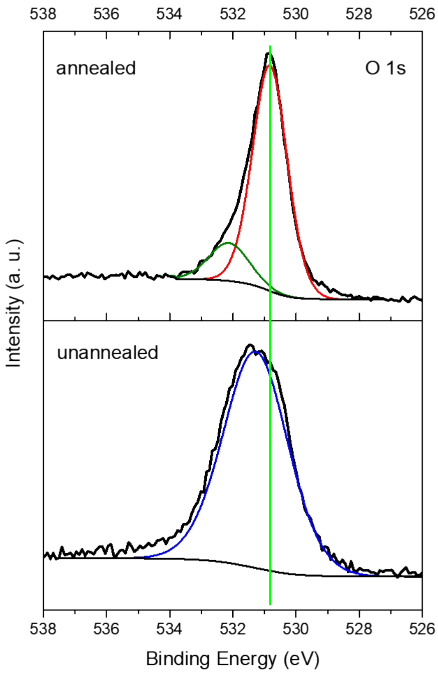

Figure S9: O 1s core level XPS spectra of substrates before and after annealing.

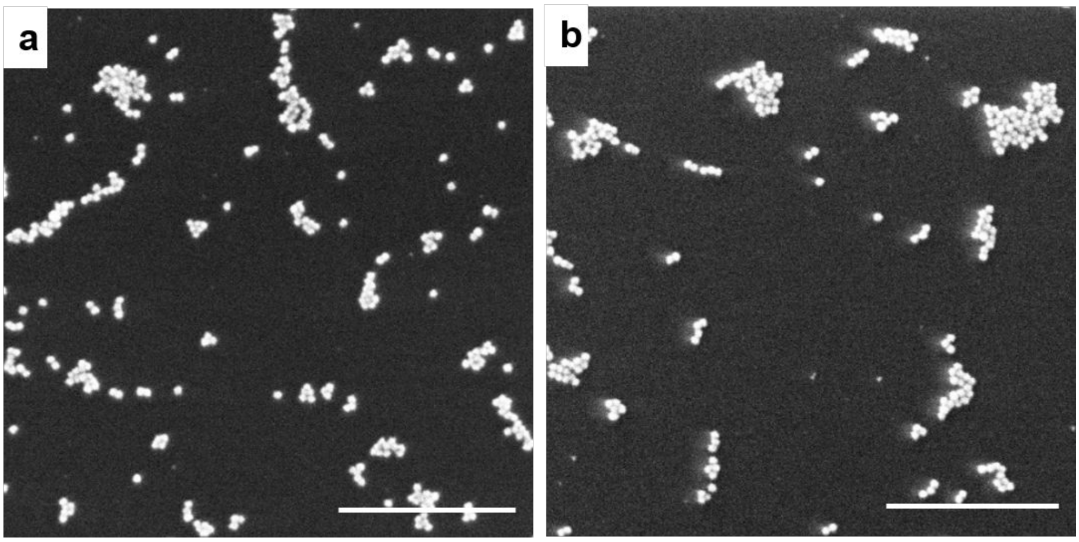

Figure S10: 28 nm PtNPs spin-coated onto degreased Si/SiO_2 substrates a) pre-anneal and (b) after annealing at 350 °C.

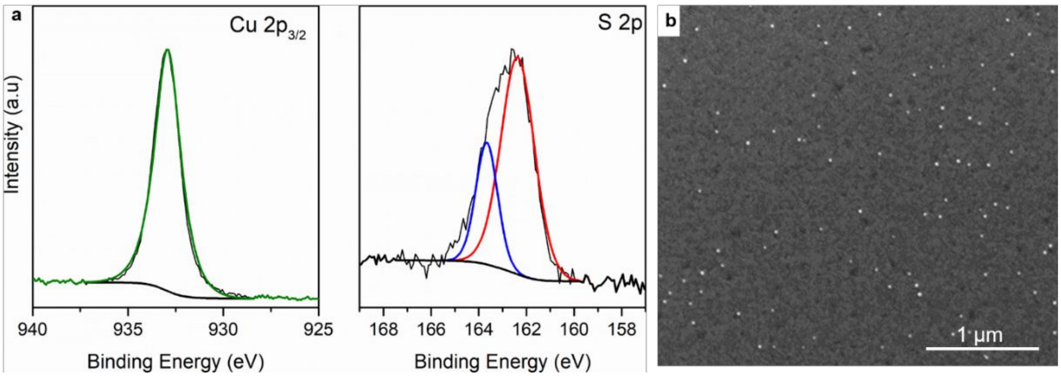

Figure S11: (a) Cu 2p and S 2p core level of Cu substrates treated with dithiol in place of diamine ligands. (b) SEM of dithiol functionalized Cu substrate after PtNP immobilized.

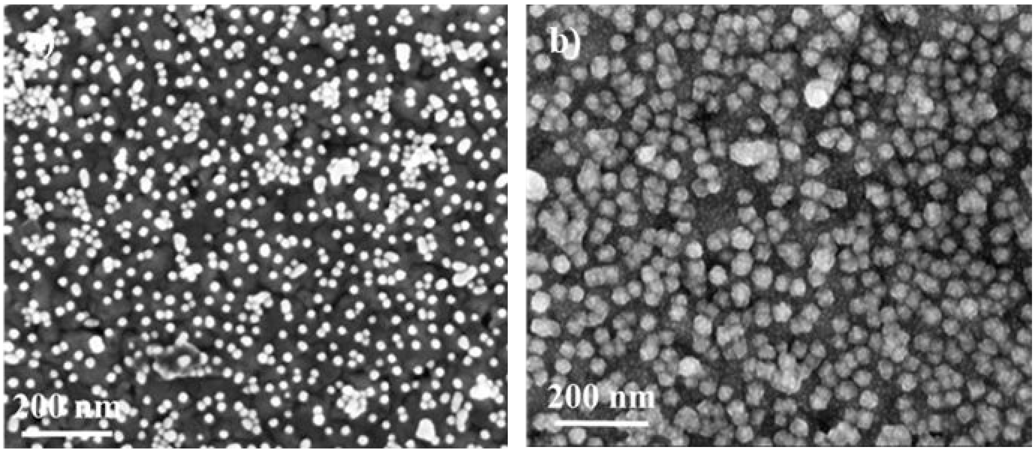

Figure S12: SEM images of Au nanoparticles supported on Cu substrates (a) unannealed and (b) after annealing at 300 °C under H_2/Ar.

Table S1: Proposed reactions in Pt catalysed (a) glucose electro-oxidation in NaOH and (b) methanol oxidation reduction under alkaline conditions.

**(a) Glucose electro-oxidation under basics conditions**
$2\,Cu^0 + 2\,OH^- \rightarrow Cu_2O + H_2O + 2\,e^-$
$Cu_2O + 2\,OH^- \rightarrow 2\,CuO + H_2O + 2\,e^-$
$Cu_2O + 2\,OH^- + H_2O \rightarrow 2\,Cu(OH)_2 + 2\,e^-$
$Cu(II) \rightarrow Cu(III) + e^-$
$Cu(III) + 4\,OH^- \rightarrow Cu(II) + 2H_2O + O_2$

$Cu(II) - glucose \underset{e}{\rightleftarrows} Cu(III) - glucose \rightarrow Gluconolactone + Cu(II)$
**(b) Methanol oxidation reduction**
**Dehydrogenation / oxidative chemisorption**
$Pt + CH_3OH = Pt\text{-}CH_3O_{ads} + H^+ + e$<br>$Pt\text{-}CH_3O = Pt\text{-}CHO_{ads} + 2H^+ + 2e$
**Poisoning**
$Pt\text{-}CHO_{ads} = Pt\text{-}CO_{ads} + H^+ + e$<br>Reactive pathway (M could represent Pt, a second metal or reduced metal oxide):<br>$M + H_2O = M\text{-}OH_{ads} + H^+ + e$<br>$Pt\text{-}CHO_{ads} + M\text{-}OH_{ads} = Pt\text{-}COOH_{ads} + M + H^+ + e$<br>$Pt\text{-}COOH_{ads} = Pt + CO_2 + H^+ + e$
**Poison removal**
$M + H_2O = M\text{-}OH_{ads} + H^+ + e$<br>$Pt\text{-}CO_{ads} + M\text{-}OH_{ads} = Pt + M + CO_2 + H^+ + e$

Table S2: The mass activity of nanocatalysts reports from literature.

Ref. No.	Catalyst	Electrolyte	Mass activity (A/mg_{Pt})
2	PtCu mesoporous nanowires	1.0 M KOH + 1.0 M CH_3OH	0.741
3	Pt_3Cu on TiO_2	1.0 M KOH + 1.0 M CH_3OH	0.437
4	Pd_9Ru@Pt/FGN	0.5 M H_2SO_4 + 1 M CH_3OH	0.881
5	PtCo CNCs	0.5 M H_2SO_4 + 1.0 M CH_3OH	0.69
6	Pt nanoparticle- RGO-MXene nanosheet	1.0 M KOH + 1.0 M CH_3OH	7.32
3	Commercial Pt/C	1.0 M KOH + 1.0 M CH_3OH	0.300
7	PtPd NCs/C	0.1 M $HClO_4$ + 0.5 M CH_3OH	1.77
8	PtAg alloy porous nanosheets	1.0 M KOH + 1.0 M CH_3OH	3.99

**This work**	$PtCu@Cu_2O$ core shell nanocube	1.0 M KOH + 1.0 M CH_3OH	**1.656**

References

1. Bigall, N. C.; Härtling, T.; Klose, M.; Simon, P.; Eng, L. M.; Eychmüller, A., Monodisperse platinum nanospheres with adjustable diameters from 10 to 100 nm: synthesis and distinct optical properties. *Nano Lett* **2008,** *8* (12), 4588-92.
2. Saini, K.; P. Ingole, P.; Sharma Bhatia, S.; Rani, N., Rod-shaped copper (Cu, Cu2O) nano catalyst for the facile oxidation of methanol. *Advanced Materials Letters* **2018,** *9* (1), 36-41.
3. Pham, H. Q.; Huynh, T. T., Platinum–Copper Bimetallic Nanodendritic Electrocatalyst on a TiO2-Based Support for Methanol Oxidation in Alkaline Fuel Cells. *ACS Applied Nano Materials* **2021,** *4* (5), 4983-4993.
4. Li, H.; Si, Z.; Zhang, J.; Fu, Y.; Li, W., Worm-like PtCux alloys oriented by pyrolyzed alginic acid sodium with high performance in methanol electrooxidation. *International Journal of Hydrogen Energy* **2021,** *46* (45), 23225-23235.
5. Li, Z.; Jiang, X.; Wang, X.; Hu, J.; Liu, Y.; Fu, G.; Tang, Y., Concave PtCo nanocrosses for methanol oxidation reaction. *Applied Catalysis B: Environmental* **2020,** *277*, 119135.
6. Liu, C.; Chen, Z.; Rao, D.; Zhang, J.; Liu, Y.; Chen, Y.; Deng, Y.; Hu, W., Behavior of gold-enhanced electrocatalytic performance of NiPtAu hollow nanocrystals for alkaline methanol oxidation. *Science China Materials* **2020,** *64* (3), 611-620.
7. Wang, C.; Huang, Z.; Ding, Y.; Xie, M.; Chi, M.; Xia, Y., Facet-Controlled Synthesis of Platinum-Group-Metal Quaternary Alloys: The Case of Nanocubes and {100} Facets. *Journal of the American Chemical Society* **2023,** *145* (4), 2553-2560.
8. Wu, T.; Gan, M.; Ma, L.; Wei, S.; Fu, Q.; Yang, Y.; Li, T.; Xie, F.; Zhan, W.; Zhong, X., Pt-based nanoparticles decorated by phosphorus-doped CuWO4 to enhance methanol oxidation activity. *New Journal of Chemistry* **2021,** *45* (25), 11035-11041.
